# All-Fiber Configuration Laser Self-Mixing Doppler Velocimeter Based on Distributed Feedback Fiber Laser

**DOI:** 10.3390/s16081179

**Published:** 2016-07-27

**Authors:** Shuang Wu, Dehui Wang, Rong Xiang, Junfeng Zhou, Yangcheng Ma, Huaqiao Gui, Jianguo Liu, Huanqin Wang, Liang Lu, Benli Yu

**Affiliations:** 1 Key Laboratory of Opto-Electronic Information Acquisition and Manipulation of Ministry of Education, Anhui University, Jiulong Road 111#, Hefei 230601, China; shuangwu1974@163.com (S.W.); dehuiwangahu@163.com (D.W.); xiangrong_1981@163.com (R.X.); zhoujunfeng215@163.com (J.Z.); myc@ahu.edu.cn (Y.M.); benliyu@ahu.edu.cn (B.Y.); 2 Key Laboratory of Environmental Optics and Technology, Anhui Institute of Optics and Fine Mechanics, Chinese Academy of Sciences, Hefei 230031, China; hqgui@aiofm.ac.cn (H.G.); jgliu@aiofm.ac.cn (J.L.); 3 State Key Laboratory of Transducer Technology, Institute of Intelligent Machines, Chinese Academy of Sciences, 350 Shu Shang Hu Road, Hefei 230031, China; hqwang@iim.ac.cn

**Keywords:** self-mixing effect, DFB fiber laser, Laser Doppler Velocimeter (LDV)

## Abstract

In this paper, a novel velocimeter based on laser self-mixing Doppler technology has been developed for speed measurement. The laser employed in our experiment is a distributed feedback (DFB) fiber laser, which is an all-fiber structure using only one Fiber Bragg Grating to realize optical feedback and wavelength selection. Self-mixing interference for optical velocity sensing is experimentally investigated in this novel system, and the experimental results show that the Doppler frequency is linearly proportional to the velocity of a moving target, which agrees with the theoretical analysis commendably. In our experimental system, the velocity measurement can be achieved in the range of 3.58 mm/s–2216 mm/s with a relative error under one percent, demonstrating that our novel all-fiber configuration velocimeter can implement wide-range velocity measurements with high accuracy.

## 1. Introduction

The traditional Laser Doppler Velocimeter (LDV) [1,2,3] based on two beam interference have been widely applied in optical sensor and industrial areas [4,5,6,7], on account of the advantages of anti-interference, fast dynamic response, and non-contact measurement. However, there still exist many restrictions in practical application, such as complex light path and high reference beam requirement. Laser self-mixing interference (SMI) technology is a novel coherent measurement technique which has been researched and developed in many sensing and measuring fields, such as absolute distance, displacement, velocity, and vibration measurement [8,9,10,11]. SMI technology applied in LDV [12,13] is called SM-LDV, which can replace traditional LDVs on many occasions because of its compact and simple structure, easy alignment, reliability, and low cost.

In the common SM-LDV system, the laser source is usually semiconductor lasers [14,15], which have been broadly investigated as they have small size, long service life, and high optical feedback sensitivity resulting from short cavity length [16]. However, wide linewidth and multi-longitudinal mode characteristics of semiconductor lasers will lead to poor coherence and monochromaticity. Thus, more effective methods have to be developed for the purpose of obtaining preferable measurement results; for instance, employing a fiber laser as the laser source of a SM-LDV system.

Recently, fiber lasers have developed rapidly, attracting considerable attention due to a variety of potential applications in fiber communication techniques and fiber sensing systems. It has been proven that fiber laser sensors show great superiority, including wide responsive bandwidth, remote optical pumping, immunity to electromagnetic interference, interrogation ability, and the capability of being a wavelength division multiplexed along a single fiber [17,18,19,20,21]. Compared to the semiconductor laser sensing technology, fiber laser sensing technology employed in SM-LDV is a preferable choice to realize a more flexible system and get better measurement results. Fiber ring lasers applied as the laser source in SM-LDV has been studied and experimented in detail [22,23], while the system measurement range and precision still have room to grow. This is because the dense longitudinal modes spacing resulting from long cavity length of the fiber ring laser would lead to multi-longitudinal modes oscillation and mode hopping in the resonant cavity, which seriously affect the stability of output laser [24]. Therefore, a shorter cavity used in the fiber laser is apparently a superior solution to solve the problem caused by the long cavity length of fiber ring lasers. The short cavity can enlarge the space of longitudinal modes and provide stable single longitudinal mode output. In addition, the laser sensitivity to optical feedback depends on the ratio of emission level lifetime and photon lifetime [25,26]; that is, shorter laser cavity length would lead to higher sensitivity to optical feedback.

The above reasons have inspired many researchers, including us, to attempt to employ fiber lasers with a shorter cavity length, such as the distributed Bragg reflection (DBR) fiber laser as the laser source in the SM-LDV [27,28]. However, the cavity of the DBR fiber laser is formed by two Fiber Bragg Gratings (FBGs), and the laser cavity length is not short enough. Therefore, we employed the DFB fiber laser with much shorter cavity length as the laser source of the SM-LDV system. The distributed feedback (DFB) fiber laser is formed of all-fiber configuration with only one FBG to realize optical feedback and wavelength selection, which have same active region and feedback area. The cavity length of DFB fiber laser is only a few millimeters, which have high sensitivity to optical feedback. Compared to semiconductor lasers, DFB fiber lasers have further advantages, including good compatibility with optical fiber, stability of output power, a more pure optical spectrum, narrow linewidth, and inherent fiber laser advantages, which have been attractive devices for a range of applications in communications and sensing [29,30,31]. With the purpose of implementing a wider range velocity and higher accuracy measurement compared to traditional SM-LDV, the DFB fiber laser was employed as the laser source of SM-LDV in our experiment.

In this paper, a novel all-fiber configuration laser self-mixing Doppler velocimeter with the DFB fiber laser as the laser source is presented, which is called all-fiber configuration DFB-SM-LDV. In the DFB-SM-LDV system, we use π-phase shifted DFB fiber laser as the laser source, expecting to acquire higher accuracy as well as a wider velocity measurement range compared to the common SM-LDV. The theory model of the all-fiber configuration DFB-SM-LDV was built up in this report, along with detailed theoretical analysis of velocity measurement.

## 2. Theoretical Simulation

**Figure 1:**
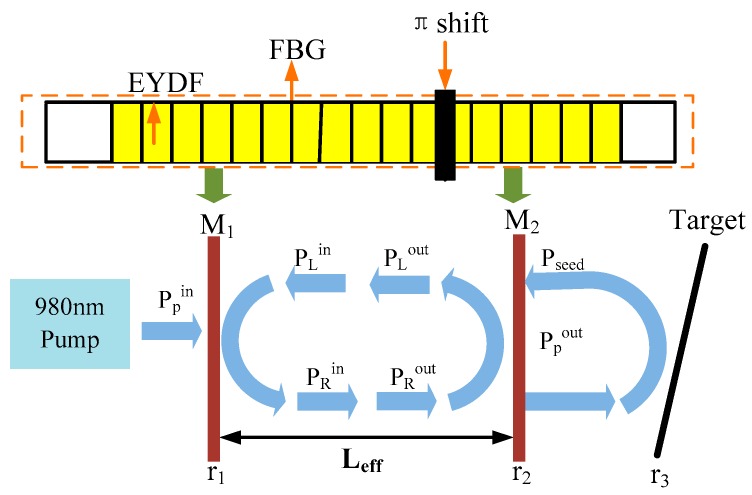
The theoretical model of all-fiber configuration self-mixing interference laser Doppler velocimeter (SM-LDV) system based on the π-phase shifted DFB fiber laser. EYDF: Er^3+^-Yb^3+^ co-doped optical fiber; FBG: fiber Bragg grating.

In this section, we proposed a basic theoretical model of DFB fiber laser based on the phase-shifted FBG. To prove the feasibility of the experiment and analyze the results of laser self-mixing output power, the theoretical model was established as shown in Figure 1. In Figure 1, the yellow part stands for the gain medium Er^3+^-Yb^3+^ co-doped optical fiber (EYDF). The black bars stand for the fiber Bragg gratings (FBG), which are written directly into the active optical fiber.

Figure 1 shows the theoretical model of the all-fiber structure DFB-SM-LDV, which is built based on the phase-shifted FBG and laser self-mixing effect. The DFB laser we employed in the experiment is a π-phase shifted DFB laser, which achieves single-wavelength operation and unidirectional output due to the extremely small length of the cavity [32,33,34]. From the phase-shift position, the original grating can be regarded as two grating sections, which can be considered as a pair of wavelength-matched FBGs. The grating section on the left side of the phase shift can be regarded as a mirror of M_1_, and the grating section on the right side of the phase shift can be regarded as the output mirror of M_2_. In the phase-shifted FBG, the fields propagating to the left and to the right are constrained by the two gratings, and they are circulating within a short effective cavity, regarded as the equivalent F-P cavity of the DFB fiber laser [35,36].

We analyze the laser self-mixing effect of the DFB fiber laser as shown in Figure 1. The pump light is coupled into the effective resonant cavity of the DFB fiber laser, the output power is focused on the object and reflected or scattered by the moving target. In the Figure 1, the P^in^, P^out^, and P_seed_ represent the input power, the output power, and the seed light power, respectively. The subscripts p and s represent the pump and signal. In addition, the subscripts L and R represent that the laser propagates to the left and right direction in the laser cavity, respectively. In the velocity measurement system, the scattered or reflected field is frequency-shifted by the Doppler principle on the moving target. By combining the quasi-analytical method to solve the steady-state equations of the lasing condition in the DFB fiber laser and the calculation of Boundary condition equations, we could theoretically deduce the expression of output power, which can be written as [37,38]:

(1) $$\begin{array}{l} P_{R}^{out}=\varepsilon_{1}^{2}r_{1}^{2}\varepsilon_{2}[\varepsilon_{2}r_{2}^{2}P_{R}^{out}+(1-r_{2}^{2})P_{seed}]\exp\left(-2\alpha_{s}L_{eff}+2P_{s}^{\mathrm{abs}}/P_{ss}+2P_{p}^{\mathrm{abs}}/P_{ss}\right)\\ =\varepsilon_{1}^{2}r_{1}^{2}\varepsilon_{2}[\varepsilon_{2}r_{2}^{2}P_{R}^{out}+(1-r_{2}^{2})P_{seed}]\exp\left\{-2\alpha_{s}\left(\frac{\tanh(\kappa L_{1})}{2\kappa }+\frac{\tanh(\kappa L_{2})}{2\kappa }\right)+2P_{s}^{\mathrm{abs}}/P_{\mathrm{ss}}+2P_{p}^{\mathrm{abs}}/P_{\mathrm{ss}}\right\} \end{array}$$

Here, P_seed_ is introduced as the total power of back-scattered light from a moving target, given as:

(2) $$P_{seed}={\left[r_{2}+\eta {\left(1-{r_{2}}^{2}\right)}^{2}r_{3}\cos\varphi_{ext}\right]}^{2}P_{R}^{out}$$

where the indexes abs and ss are powers of the absorbed in one round trip and saturation, respectively. P_p_^abs^ and P_s_^abs^ are the variable values of the pump and signal light power, respectively. P_ss_ denotes the saturation powers of signal light. α_s_ is the small signal absorption coefficient. L_eff_ is the length of the effective active doped fiber. The length of the left and right grating section are L_1_ and L_2_, respectively. κ is the coupling coefficient. *r*_1_, *r*_2_, and *r*_3_ are the reflection coefficient of the M_1_, M_2_, and the target, respectively. *η* is the coupling efficiency of the object to the collimator. ε_1_ and ε_2_ represent the total attenuation factors, including insertion loss.

When the external target moves far away from the laser, the external phase can be expressed as:

(3) $$\varphi_{ext}=\frac{4\pi \left(L_{0}+vtcos\theta \right)}{\lambda }$$

**Figure 2:**
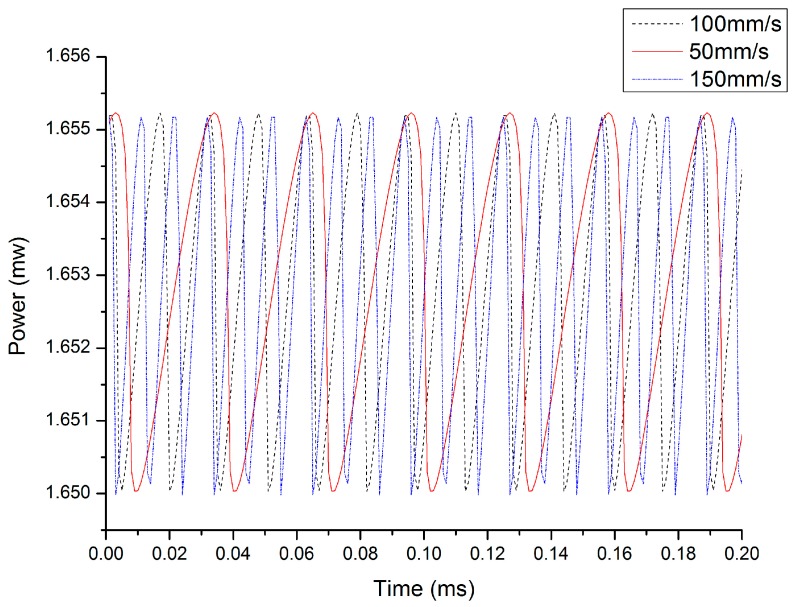
The simulated self-mixing output signals at different velocities.

Here, v is the velocity of a moving target and *θ* is the angle between the incident light direction and the velocity direction. On the basis of the above equations, a simulation of laser output power fluctuation caused by the speed of an external target was carried out. The simulated self-mixing signals at different velocities are shown in Figure 2; the red solid line, black dotted line, and blue dotted line are the self-mixing signal corresponding to the external target speed at 50 mm/s, 100 mm/s, and 150 mm/s, respectively. From Figure 2, we can see that the self-mixing signal fringe number increases linearly with increasing external target velocity.

Through a Fast Fourier Transform (FFT) of the simulated output power at a certain speed, the corresponding frequency can be obtained. In order to further explore the relationship of external target speed and corresponding frequency, we simulated the speed of the external target from 300 mm/s to 3000 mm/s, with steps of 300 mm/s at θ = 60°.

**Figure 3:**
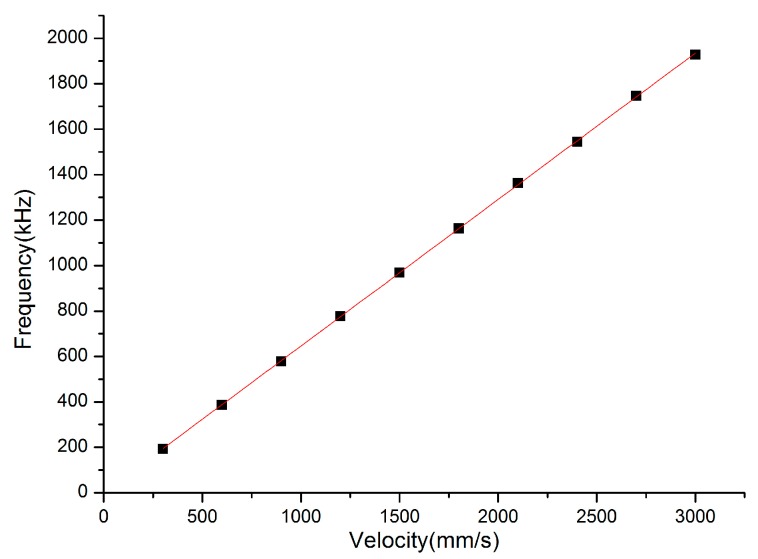
The simulated frequency of the external moving target at different velocities with θ = 60°.

The simulated results of the simulated velocity and corresponding frequency are plotted in Figure 3, which are indicated by dots and fitted by a red line. From Figure 3, we can observe that the relationship between the simulated velocity and corresponding frequency is in accordance with the Doppler principle, which indicates that it is a considerable method to utilize the self-mixing signal frequency to measure the velocity of a moving target.

## 3. Schematic of the Experimental Set-up

**Figure 4:**
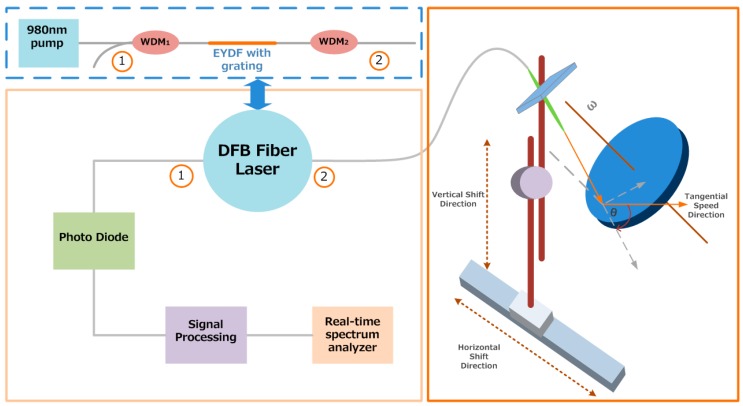
The experimental set-up of our novel all-fiber configuration SM-LDV based on the distributed feedback (DFB) fiber laser.

For the sake of verifying the feasibility of utilizing the laser self-mixing Doppler principle to measure velocity, experiments have been carried out. The experimental setup is shown schematically in Figure 4. In our experimental system, the all-fiber configuration DFB-SM-LDV consists of a DFB fiber laser, a target consisting of a rotating turntable with a rough reflective surface, and a signal processing circuit with a photo-diode (PD). As shown in Figure 4, the pump light is coupled to the effective cavity of the DFB fiber laser through wavelength division multiplex (WDM1), and the signal is amplified by the gain medium. The output laser is scattered by the moving turntable and is reflected back into the effective cavity of the DFB fiber laser, mixing with the initial laser, modulating the output power and spectrum.

The DFB fiber laser is made up of two 980 nm/1550 nm WDMs and gain medium of active optical fiber with phase-shifted FBG, which is used to realize optical feedback and wavelength selection. The lasing action in the DFB laser can be considered as signal generation by the gain medium and feedback by the FBG. The gain medium of the DFB fiber laser we used is Er^3+^-Yb^3+^ co-doped optical fiber (EYDF), and the phase shifted FBG had been written in it directly. The reason for employing EYDF as the gain medium is that the DFB fiber laser requires high pump efficiency, owing to the short cavity length. EYDF has higher gain, wider range of pump light wavelength, as well as higher pump efficiency compared to commonly Er^3+^ doped fiber, which can satisfy the need of high pump efficiency. Thus, employing EYDF as the gain medium in the DFB fiber laser is an efficient way to solve the issues of low pump efficiency.

As shown in Figure 4, the target is a rotating turntable with a rough reflective surface which is driven by a DC motor (Chongqing, China, Feiteng 41K2.5RGN-C direct current motor). The rotational speed of the turntable is controlled by the motor’s speed controller, with the revolving speed range of 0–3.6 rotations per second. The laser output is through a collimator fitted onto the three-dimensional adjustable support brackets, which is employed to adjust the angle (θ) between the incident light and the measured velocity, as well as to regulate the collimator in vertical and horizontal shift directions to ensure the velocity at a certain point of the turntable. Then, the collimator receives the light reflected and scattered from the surface of the turntable. The modulated output power is detected by the PD connected at the end of the processing circuit, which consists of an amplifying and a filtering circuit. The received signal is processed to realize signal amplifying and noise reduction by the signal processing circuit. The processed signal is observed on a radio-frequency (RF) spectrum analyzer (Ohio, America, Tektronix RSA-3408B real time spectrum analyzer).

It can be seen that the laser incident upon a certain point of the turntable and the tangential speed direction of that point is shown in Figure 4, which can be projected into the light incident direction and perpendicular to the direction of the incident light. In our experiment, the value of the angle between the incident light and the velocity is 60°. Additionally, in our experiment, the turntable rotates at a fixed direction, so the phase direction remains unchanged [39,40]. The tangential velocity of a certain point on the turntable is determined by the rotating speed of the turntable and the distance between that point and the turntable center.

## 4. Experimental Results and Discussion

**Figure 5:**
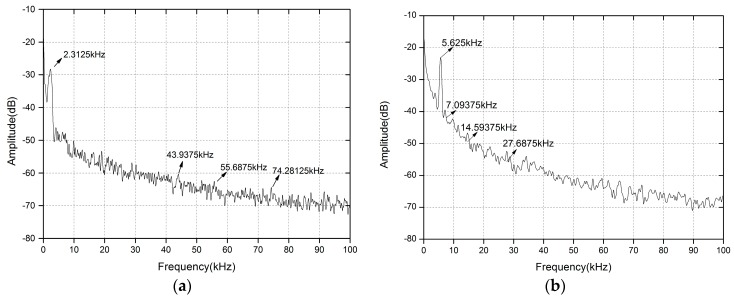
Typical Doppler signal displayed on the spectrum analyzer of different velocities with θ = 60°. (**a**) v = 3.585 mm/s; (**b**) v = 8.724 mm/s.

**Figure 6:**
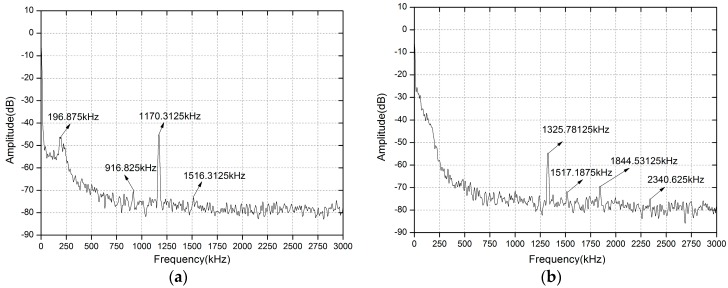
Typical Doppler signal displayed on the spectrum analyzer of different velocities with θ = 60°. (**a**) v = 1814.88 mm/s; (**b**) v = 2053.68 mm/s.

In the experiment, by adjusting the rotating speed of the turntable and changing the incident position of the laser, we can continuously measure velocities ranging from 3.58 mm/s to 2216 mm/s. A spectrum analyzer (Tektronix RSA-3408B real time spectrum analyzer) is employed to observe the spectrum related to the measured velocity. Through observing the spectrum analyzer, the Doppler frequency of the certain point of the turntable can be acquired. We give typical Doppler signals displayed on the frequency spectrum analyzer in the small velocity measurement range and large velocity measurement range, which are shown in Figure 5 and Figure 6. In Figure 5, we can observe the experimental result of the all-fiber configuration DFB-SM-LDV under the condition of θ = 60° at a velocity of 3.585 mm/s and 8.724 mm/s, respectively. As shown in Figure 5, the values of the Doppler frequency peaks caused by the measured velocities are 2.3125 kHz and 5.625 kHz, respectively.

Figure 6 shows the experimental results of the all-fiber configuration DFB-SM-LDV affected by the turntable under the condition of θ = 60° at a velocity of 1814.88 mm/s and 2053.68 mm/s, respectively. From Figure 6, we can see that the values of the Doppler frequency peaks caused by the measured velocities are 1170.3125 kHz and 1325.78125 kHz, respectively.

From the data shown in Figure 5 and Figure 6, there are some small extra peaks (which have been remarked) corresponding to the frequencies of environmental vibration signal and electronic noise signal. Meanwhile, it can be seen that the Doppler signals have certain broadening, which is derived from laser spectral broadening and non-uniform velocity distribution such as the inhomogeneity of angular speed, the inconformity of speed of each point in the irradiation light spot, the determination of turntable center position, and the speckle effect. In our experimental system, we adopt the peaking searching method to collect the frequency peak value. Based on the frequency peak value, we could calculate the corresponding velocity according to the Doppler principle, which was clearly introduced in the theoretical simulation. This kind of peak searching method can mainly reduce the influence of sideband and broadening of the Doppler signals, but the uncertainty on any peak position still depends on its width and the acquisition’s horizontal resolution (Resolution Bandwidth of the Spectrum Analyzer changes from 300 Hz to 10 KHz with different frequency-scales) and vertical resolution (1 dB).

**Figure 7:**
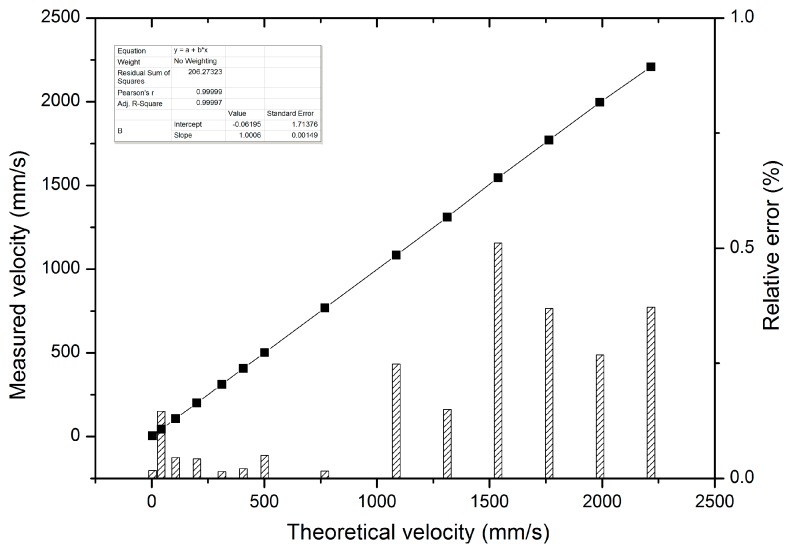
The relative error of measured velocity and theoretical velocity.

The experimental results of the measured velocity of the external target are shown in Figure 7. As shown in Figure 7, black spots represent the measured velocities and the shadow bar represents the relative error of the all-fiber DFB-SM-LDV system. The velocity measurement range is from 3.58 mm/s to 2216 mm/s. The minimum measurement velocity in our experiment is 3.58 mm/s, which is lower than the minimum measurement result of SM-LDV with DBR fiber laser and ring laser in prior velocity measurement works [22,27], proving that our novel all-fiber configuration DFB-SM-LDV has a much wider measurement range compared to the SM-LDV with DBR fiber laser and ring laser. For the moment, confined to mechanical conditions in the experiment, such as the minimum rotation speed and the dimension of the turntable, the velocity measurement range is limited. In order to realize a much wider-range and higher-precision velocity measurement, the experimental set-up should be improved and optimized repeatedly in further work.

From Figure 7, we can see that the relative error is less than one percent in the whole experimental range, which satisfies the requirement of high-accuracy measurement. The measured error may be introduced by the rotating turntable; the reason is that when the turntable rotates at a high speed, some vibration would be inevitable, which will cause the turntable rotational speed to be uneven and finally influence the measurement results. In the experiment, the measurement precision is increased by averaging the testing values of repeated measurements. In addition, we used the method of peak searching instead of the traditional fringe counting method to seek the corresponding Doppler frequency of the measured velocity, which can increase the measurement accuracy and avoid measurement error stemming from signal broadening.

As shown in the above theoretical analysis and experimental results, it can be observed that the all-fiber configuration DFB-SM-LDV has many advantages as well as wide measurement range and high precision, which can solve the problems of small measurement range, complex structure, and low measurement accuracy caused by other LDVs.

## 5. Conclusions

In conclusion, this paper presents a particular description of theoretical and experimental studies of an all-fiber DFB fiber laser self-mixing Doppler velocity measurement system, which has been proposed for the first time. In the velocity measurement system, the scattered field is frequency-shifted by the Doppler principle on the moving target, and the Doppler frequency is linearly proportional to the value of velocity, which was theoretically analyzed and experimentally observed. In our experiment, the range of velocity measurement is between 3.58 mm/s and 2216 mm/s, and the relative error between the measured velocity and the actual velocity is under one percent. From the experimental results, it can be concluded that our novel all-fiber configuration DFB-SM-LDV system can achieve high-precision, wide-range velocity measurement, which has a great potential for a number of practical applications, such as high-speed measurement and velocity measurement in common room conditions without accurate environmental control.
